# Fatty acid feedstocks enable a highly efficient glyoxylate‐TCA cycle for high‐yield production of β‐alanine

**DOI:** 10.1002/mlf2.12006

**Published:** 2022-06-19

**Authors:** Yingchun Miao, Jiao Liu, Xuanlin Wang, Bo Liu, Weifeng Liu, Yong Tao

**Affiliations:** ^1^ CAS Key Laboratory of Microbial Physiological and Metabolic Engineering, State Key Laboratory of Microbial Resources, Institute of Microbiology Chinese Academy of Sciences Beijing China; ^2^ College of Life Science University of Chinese Academy of Sciences Beijing China; ^3^ Microcyto Co. Ltd. Beijing China

**Keywords:** β‐alanine, fatty acid feedstocks, glyoxylate shunt, TCA cycle, *Escherichia coli*

## Abstract

Metabolic engineering to produce tricarboxylic acid (TCA) cycle‐derived chemicals is usually associated with problems of low production yield and impaired cellular metabolism. In this work, we found that fatty acid (FA) feedstocks could enable high‐yield production of TCA cycle‐derived chemicals, while maintaining an efficient and balanced metabolic flux of the glyoxylate‐TCA cycle, which is favorable for both product synthesis and cell growth. Here, we designed a novel synthetic pathway for production of β‐alanine, an important TCA cycle‐derived product, from FAs with a high theortecial yield of 1.391 g/g. By introducing *panD*, improving *aspA,* and knocking out *iclR*, glyoxylate shunt was highly activated in FAs and the yield of β‐alanine reached 0.71 g/g from FAs, much higher than from glucose. Blocking the TCA cycle at *icd/sucA/fumAC* nodes could increase β‐alanine yield in a flask cultivation, but severely reduced cell growth and FA utilization during fed‐batch processes. Replenishing oxaloacetate by knocking out *aspC* and recovering *fumAC* could restore the growth and lead to a titer of 35.57 g/l. After relieving the oxidative stress caused by FA metabolism, β‐alanine production could reach 72.05 g/l with a maximum yield of 1.24 g/g, about 86% of the theoretical yield. Our study thus provides a promising strategy for the production of TCA cycle‐derived chemicals.

## INTRODUCTION

Microbial cell factory provides a promising approach to producing high value‐added chemicals from renewable feedstocks with minimized environmental impact. To develop an efficient cell factory, the central metabolism is usually rewired to direct metabolic flux toward product synthesis. This approach, however, is often accompanied by impaired cellular metabolism. The tricarboxylic acid (TCA) cycle is one of the most important central metabolic pathways. It can provide precursors for synthesizing various essential cellular components, such as amino acids and nucleotides. Concerning the industrial importance, diverse high value‐added chemicals are produced from TCA cycle intermediates, including succinate, itaconate, fumarate, L‐aspartate, malate, and β‐alanine^[^ ^1^, ^2^, ^3^, ^4^ ^]^. However, it is still challenging to develop a strategy for the efficient supply of TCA cycle intermediates.

Theoretically, the most efficient pathway to supply TCA cycle intermediates is through the phosphoenolpyruvate carboxylase (PEPC) anaplerotic pathway, where TCA cycle intermediates are replenished through the carboxylation of phosphoenolpyruvate (PEP) or pyruvate. Previously, we developed an efficient route to produce L‐aspartate and β‐alanine through the PEPC anaplerotic pathway, and a yield of 0.425 g/g glucose was achieved^[^ ^5^ ^]^. However, the yield still could not reach the theoretical yield. This is mainly because a part of the metabolic flux is diverted to the TCA cycle, which is necessary for energy generation to support cellular functions. Furthermore, the glycolysis pathway of the engineered strain was disrupted so as to maximize the metabolic flux toward oxaloacetate (OAA), which might lead to a reduction in cell growth during fermentation^[^ ^5^, ^6^ ^]^. An alternative pathway to supply the TCA cycle intermediates is through the glyoxylate shunt and the oxidative branch of the TCA cycle. This route works under aerobic conditions, making it easily applicable for industrial‐scale production. However, this route requires acetyl‐CoA as the precursor, which is associated with decreased yield due to the loss of carbon during acetyl‐CoA formation from pyruvate. Furthermore, when using glucose as feedstock, metabolic flux diversion exists at several nodes within glycolysis pathway, posing a challenge to metabolic control. Another drawback lies in the fact that glucose has a relatively lower carbon content per mass (40%) and is usually associated with low molecular efficiency in the biosynthesis route^[^ ^7^, ^8^ ^]^, which further limits the theoretical yield of chemicals and decreases the process efficiency^[^ ^9^, ^10^, ^11^ ^]^.

**Figure 1:**
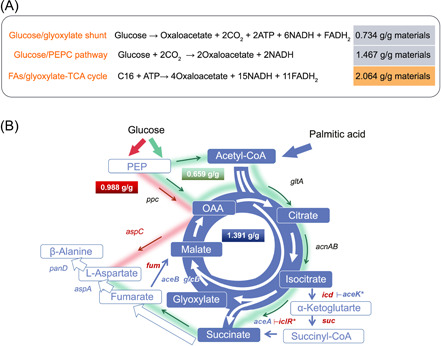
FA feedstocks enable a higher yield of TCA derivative production than glucose. (A) Comparison of metabolic pathway routes of bioproduction of C4 tricarboxylic acid (TCA) cycle intermediates (OAA) from FA or glucose feedstocks. Theoretical yields of each route are shown. (B) Design of glyoxylate‐TCA cycle for efficient production of TCA cycle‐derived chemicals. Genes and their encoded enzymes: *aceA*: isocitrate lyase; *gltA*: citrate synthase; *aceB/glcB*: malate synthase; *icd*: isocitrate dehydrogenase; *suc*; 2‐oxoglutarate dehydrogenase complex; *fum*: fumarase; *aspA*: aspartate ammonia‐lyase; *iclR*: glyoxylate bypass operon transcriptional repressor; *panD*: aspartate α‐decarboxylase; *aceK*: isocitrate dehydrogenase kinase/phosphatase; *aspC*: aspartate aminotransferase; *acnAB*: aconitate hydratase; *ppc*: phosphoenolpyruvate carboxylase. Blue flowchart indicates the β‐alanine production route from FA feedstocks through the glyoxylate‐TCA cycle. Green flowchart indicates the β‐alanine production route from glucose feedstock through the glyoxylate shunt. Red flowchart indicates the β‐alanine production route from glucose feedstock through the PEPC pathway. Genes in red color indicate deletion of the corresponding gene. Genes in blue color indicate overexpression of the relevant genes through plasmid‐based expression or promoter replacement.

Fatty acids (FAs) are promising alternative feedstocks to glucose and can be easily obtained from various sources, including waste oil, crude oil, and oil by‐products^[^ ^12^, ^13^, ^14^, ^15^ ^]^. The metabolic profiles of FAs are far different from glucose. For example, glyoxylate shunt is highly active to promote FA metabolism during seed germination in some plants^[^ ^16^ ^]^. In our previous work, we found the TCA cycle can be significantly activated when using FAs as feedstocks in an engineering *Escherichia coli* strain^[^ ^17^ ^]^. We, therefore, speculated FA feedstocks might be suitable for the production of TCA cycle‐derived chemicals due to favorable metabolic regulation. FA catabolism can generate abundant acetyl‐CoA serving as the precursor of TCA cycle intermediates. Meanwhile, since acetyl‐CoA is directly generated by β‐oxidation of FAs, the metabolic control will be more straightforward and convenient. In addition, FAs have a higher degree of reduction and carbon content per mass (75%) compared to glucose. Thus, their catabolism allows generation of more reducing equivalents required for biosynthesis and can produce chemicals with higher yield. For example, when producing TCA cycle intermediate OAA from palmitic acid (C16 FA) feedstocks, the theoretical yield is 2.064 g/g materials, higher than that of both PEPC route (theoretical yield of 1.467g/g materials) and glyoxylate shunt route (theoretical yield of 0.734 g/g materials) from glucose feedstock (Figure 1A). Furthermore, large amounts of energy and reducing power generated in this route could also facilitate cell growth and improve pathway efficiency (Figure 1A).

In this study, we sought to construct FA feedstocks‐based glyoxylate‐TCA route to achieve highly efficient supply of TCA cycle intermediates. A pathway for producing β‐alanine from FAs was designed. β‐Alanine is an important nonprotein amino acid derived from TCA cycle intermediates. It can be used as precursors for producing pantothenic acid^[^ ^18^ ^]^, acrylamide, and acrylonitrile^[^ ^19^ ^]^, which are widely applied in the production of food additives, pharmaceuticals^[^ ^20^ ^]^, and bulk chemicals. Previously, two metabolic routes of β‐alanine production from glucose have been developed. By using glyoxylate shunt under aerobic and PEPC pathway under microaerobic conditions, β‐alanine yields can reach 0.135 g/g glucose^[^ ^6^ ^]^ and 0.425 g/g glucose^[^ ^5^ ^]^, respectively (Table 1). Here, we proposed β‐alanine could be produced from FAs with high theoretical yield. The precursor, OAA or fumarate, could be obtained from the glyoxylate‐TCA cycle, and the theoretical yield reached 1.391 g/g raw materials (palmitic acid), which is much higher than any route from glucose (Figure 1).

To develop an efficient metabolic route to produce TCA cycle‐derived chemicals from FAs, some issues should be considered. First, cells undergo a different regulation on the glyoxylate shunt and the TCA cycle in FAs. The metabolic profiles of the two pathways under FA‐utilization conditions should be systematically investigated^[^ ^23^, ^24^, ^25^ ^]^. Another challenge is establishing an efficient FA bioconversion process at a large scale. When developing an efficient bioconversion strategy, different factors should be considered, including cell growth, FA utilization, energy metabolism, and β‐alanine biosynthesis. The metabolic flux under large‐scale conditions should be explored. In this study, we systematically balanced and optimized the metabolic flux between the TCA cycle, glyoxylate shunt and β‐alanine biosynthesis pathway at different levels. An efficient route for β‐alanine production from FA feedstocks was thus developed. We anticipate that the principles of TCA cycle regulation elucidated here would provide insight into developing novel bioproduction routes in the future.

## RESULTS

### FA feedstocks enable a higher yield of β‐alanine production than glucose through the glyoxylate‐TCA cycle

A biosynthetic route for β‐alanine production with a high theoretical yield was designed through the glyoxylate‐TCA cycle with FAs as feedstocks. The theoretical yield of this route could reach 1.391 g/g when using palmitic acid (C16) as the raw material, and 1.433 g/g when using tripalmitin as raw material, which is much higher than those of the routes from a glucose feedstock (through the glyoxylate shunt + PEPC [0.659 g/g] or PEPC pathway [0.988 g/g]) (Figure 1B).

**Figure 2:**
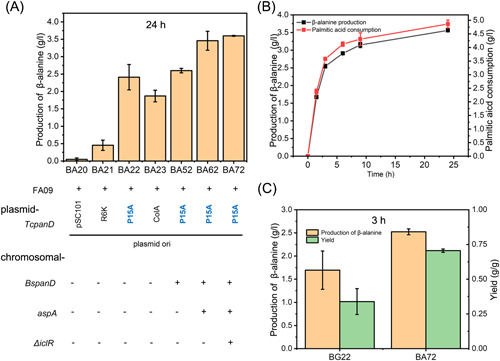
Development of β‐alanine production strains from palmitic acid. (A) β‐Alanine production was improved by the enhancement of *panD* and *aspA,* and knocking out *iclR*. L‐aspartate‐α‐decarboxylase encoded by *panD* with different copy numbers was introduced into the FA‐using chassis FA09. The genotype of each strain is shown. (B) Time curve of β‐alanine production by strain BA72. Black line indicates β‐alanine production. Red line indicates palmitic acid consumption. (C) Comparison of production and yield at 3 h between BA72 and BG22, using palmitic acid and glucose feedstocks, respectively. β‐Alanine was produced using glucose uptake modified strain BG22 from glucose feedstocks or strain BA72 from palmitic acid feedstocks. Error bars show standard deviation (*n* = 3).

To verify the efficiency of this new route, chassis strains for β‐alanine production from FA feedstock were developed by introducing L‐aspartate‐α‐decarboxylase encoded by *panD* into the FA‐using chassis FA09. *PanD* from *Tribolium castaneum* (*TcpanD*) was expressed using plasmid pSB1K (pSC101 origin)^[^ ^26^ ^]^. The resulting strain BA20 could produce β‐alanine at a concentration of 0.047 ± 0.04 g/l after bioconversion for 24 h. The expression of *TcpanD* was further optimized using different plasmids and the high β‐alanine production of 2.40 ± 0.37 g/l was obtained using the medium copy number plasmid pXB1K (p15A origin, strain BA22) (Figure 2A). When additional *panD* from *Bacillus subtilis* (*BspanD*) was chromosome‐expressed under the P119 promoter, the resulting strain BA52 could produce β‐alanine with 2.60 ± 0.06 g/l after bioconversion for 24 h (Figure 2A). The supply of L‐aspartate is another major limiting step of the β‐alanine biosynthesis pathway. We chromosome‐expressed the aspartate ammonia‐lyase encoded by *aspA* catalyzing L‐aspartate production from fumarate, which resulted in β‐alanine production of approximately 3.46 ± 0.27 g/l (strain BA62) (Figure 2A). Plasmid expression of *aspA* could not improve β‐alanine production (Figure S1).

In this biosynthetic route, a central task is to maximize the metabolic flux of the glyoxylate‐TCA cycle. We first knocked out the repressor *iclR* to activate the glyoxylate shunt. However, after *iclR w*as knocked out, the resulting strain BA72 could produce 3.60 ± 0.01 g/l of β‐alanine, only slightly increased compared with BA62 (Figure 2A). To test if FAs are better feedstocks than glucose for β‐alanine production, the production of β‐alanine was compared between BA72 and BG22, a β‐alanine producing strain differing from BA72 by using glucose as substrate instead of FAs. The production parameters were determined after bioconversion for 3 h (corresponding to the fast β‐alanine production phase, Figure 2B). It was shown both titer and yield of β‐alanine in FAs were much higher than those in glucose. The β‐alanine production from FAs was 2.52 ± 0.06 g/l (strain BA72), while from glucose was only 1.69 ± 0.41 g/l (strain BG22) (Figure 2C). The yield of β‐alanine from FA‐using strain BA72 was approximately 0.71 ± 0.01 g/g, which was much higher than the yield of 0.33 ± 0.09 g/g from glucose (BG22) (Figure 2C).

### Glyoxylate‐TCA cycle is highly upregulated under FA condition

Because the yield of β‐alanine production was still low, approximately 50% of its theoretical value, we then carried out experiments to determine the factors that limited the yield of β‐alanine. First, we speculated that acetyl‐CoA generated from β‐oxidation of FAs might not efficiently enter the TCA cycle. However, no accumulation of acetate, L‐lactate, or other acetyl‐CoA derived by‐products was observed during BA72 bioconversion.

**Figure 3:**
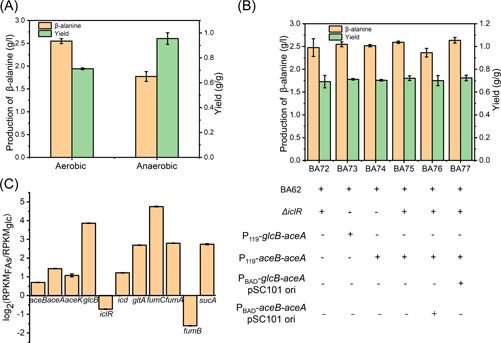
Identification of the bottleneck of β‐alanine yield. (A) β‐Alanine production and yield in aerobic and anaerobic conditions. The β‐alanine production and yield were determined at 3 h using palmitic acid feedstocks. (B) β‐Alanine production and yield by modifying the glyoxylate shunt targets. The β‐alanine production and yield were determined at 3 h using palmitic acid feedstocks. (C) mRNA levels of glyoxylate shunt genes of FA09 strain when palmitic acid or glucose was used as feedstock. *aceB*: malate synthase A; *aceA*: isocitrate lyase; *aceK*: isocitrate dehydrogenase kinase; *glcB*: malate synthase G; *iclR*: glyoxylate bypass operon transcriptional repressor; *icd*: isocitrate dehydrogenase; *fumA*: fumarase A; *fumC*: fumarase C; *fumB*: fumarase B; *sucA*: 2‐oxoglutarate dehydrogenase E1 component; *gltA*: citrate synthase; RPKM_FAs_: Reads Per Kilobase per Million mapped read in FA feedstocks; RPKM_glc_: Reads Per Kilobase per Million mapped read in glucose feedstock. Error bars show standard deviation (*n* = 3).

Thus we mainly focused on the metabolic balance between the glyoxylate shunt and the TCA cycle. We speculated that the oxidative steps of the TCA cycle would compete with the glyoxylate shunt for the metabolic flux, leading to carbon loss and thus low product yield. A notable clue was that the yield of β‐alanine production under anaerobic conditions could reach nearly 1.00 g/g, about 34% higher than that under aerobic conditions, even though the titer of β‐alanine was lower (Figure 3A). This result was in line with the fact that the activities of the oxidative branch of TCA cycle enzymes were downregulated under oxygen‐limited conditions^[^ ^27^, ^28^ ^]^. Therefore, we hypothesized that the major bottleneck of β‐alanine yield was the flux distribution between the TCA cycle and glyoxylate shunt at the isocitrate node. We first carried out several modifications to improve the expression of glyoxylate shunt enzymes to direct metabolic flux toward the glyoxylate‐TCA cycle. In addition to *iclR* being knocked out, the expression of *aceA*, *aceB*, *aceK*, and *glcB* was fine‐tuned by either replacement of their native promoters or heterologous expression. Unfortunately, no apparent improvement in β‐alanine production was observed (Figure 3B). As shown in our transcriptional data, the mRNA levels of the glyoxylate shunt genes were significantly upregulated when FAs were used as feedstocks (Figure 3C). This result was also in agreement with our previous report that the TCA cycle was activated under FA feedstocks conditions^[^ ^17^ ^]^. These results clearly indicated that the upregulation of glyoxylate shunt is an autonomous response to FA feedstocks condition and thus results in a favorable metabolic flux toward glyoxylate‐TCA cycle and β‐alanine biosynthesis. Nevertheless, additional modifications are still needed to improve the metabolic flux through the glyoxylate‐TCA cycle.

### Block of TCA cycle can improve β‐alanine yield in the flask cultivation but impede cell growth in the fed‐batch cultivation

According to the above results, blocking the oxidative branch of the TCA cycle might be necessary to improve the metabolic flux toward glyoxylate shunt. In the β‐alanine biosynthesis pathway, there are two routes for the supply of L‐aspartate, one catalyzed by L‐aspartate aminotransferase (AspC) using OAA as substrate and L‐glutamate as amino group donor, the other one catalyzed by AspA using fumarate as substrate and ammonia as amino group donor. The blocking strategies were designed based on either of the two L‐aspartate supply routes. First, we focused on SucA as the target. As indicated in our previous study, nitrogen metabolism significantly influenced FAs utilization^[^ ^17^ ^]^. Knocking out *sucA* rather than *icd* will be beneficial to the biosynthesis of L‐glutamate/L‐glutamine, which plays central regulation roles in FA utilization. L‐Glutamate is also an important cofactor of L‐aspartate aminotransferase (AspC) during β‐alanine biosynthesis. It is expected that the β‐alanine biosynthesis efficiency could be improved by blocking the TCA cycle at the *sucA* node and using *aspC* for L‐aspartate supply.

**Figure 4:**
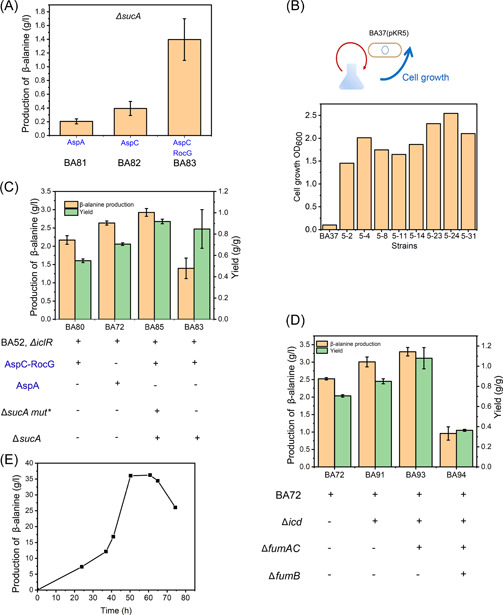
Engineering of the TCA cycle to improve the β‐alanine yield. (A) β‐Alanine production in *sucA* knocked‐out strains. BA81 with AspA overexpressed, BA82 with AspC (L‐aspartate aminotransferase) overexpressed, and BA83 with AspC‐RocG (NADH‐dependent L‐glutamate dehydrogenase) overexpressed were used, β‐alanine production was determined at 3 h using palmitic acid as feedstocks. (B) Adaptive laboratory evolution of *sucA* knocked‐out strain. Plasmid pKR5 was used for genome evolution. M9 salt medium with palmitic acid as feedstocks was used for strain growth. X‐axis represents different screened strains. (C) β‐Alanine production and yield of *sucA* knock‐out and mutant strains. β‐Alanine production was determined after whole‐cell bioconversion for 3 h using palmitic acid as feedstocks. The genotype of each strain is shown. Overexpressed enzymes are indicated in blue color. (D) β‐Alanine production in *aspA*‐*icd* strategy. β‐Alanine production was determined after whole‐cell bioconversion for 3 h using palmitic acid as feedstocks. The genotype of each strain is shown. (E) Bioconversion of BA93 at 1 l fed‐batch level. Two‐stage bioconversion strategy was used and the process was described in Materials and Methods section. Error bars show standard deviation (*n* = 3).

When *sucA* gene was knocked out, the cell growth of the resulting strain BA37 was significantly reduced (data not shown). The β‐alanine production in corresponding strain BA81 was decreased at a very low level when using *aspA* (Figure 4A). When AspA was replaced by AspC (BA82), especially when NADH‐dependent L‐glutamate dehydrogenase RocG from *B. subtilis* was also introduced to regenerate cofactor L‐glutamate, as expected, β‐alanine production could be restored to about 1.40 ± 0.28 g/l (BA83, Figure 4A), and the yield of β‐alanine production was increased to 0.85 ± 0.18 g/g, higher than BA72 (Figure 4C). However, 1.01 ± 0.06 g/l acetate was accumulated after bioconversion for 24 h (Figure S2). To resolve the problem of low cell growth of Δ*sucA* strains, we then carried out adaptive laboratory evolution using the GREACE method to improve the cell property of BA37 (Figure 4B). After nine generations of evolution, an evolved strain BA38, which had a high cell growth rate and still retained all genotypes, was obtained (data not shown). The corresponding β‐alanine producing strain BA85 could produce β‐alanine up to 2.90 ± 0.11 g/l, while the yield reached 0.92 ± 0.02 g/g (Figure 4C), about 67% higher than that with the non‐Δ*sucA* strain BA80. And the production of acetate was decreased from 1.01 ± 0.06 to 0.56 ± 0.04 g/l (Figure S2).

We also optimized the alternative strategy in which the TCA cycle was blocked at the *icd* node. In an *icd* knocked‐out strain BA91 with AspA being used for L‐aspartate supply, β‐alanine production was shown to be approximately 3.00 ± 0.14 g/l (3 h), and the yield of BA91 reached 0.85 ± 0.02 g/g (Figure 4D). No acetate accumulation was observed. Differing from our previous speculation, the β‐alanine yield of the *aspC*‐Δ*sucA* (BA85) route was just similar to that of the *aspA*‐Δ*icd* route (BA91) (Figure 4D). As the substrate of AspC, OAA also served as a key intermediate to maintain the TCA cycle, making it difficult to manipulate for further optimization. Thus, by comparing these two routes, we hypothesized that the *aspA*‐Δ*icd* route is a better choice for β‐alanine production.

Indeed, the β‐alanine yield in *aspA*‐Δ*icd* strains can be further increased by blocking the flux from fumarate toward L‐malate by knocking out *fumAC*. The β‐alanine production of BA93 was about 3.29 ± 0.12 g/l (3 h), and the yield could reach up to 1.08 ± 0.11 g/g (Figure 4D). Surprisingly, further knock out of *fumB* in strain BA94 significantly reduced the β‐alanine production to 0.95 ± 0.19 g/l (Figure 4D) and increased the acetate level to 1.6 ± 0.01 g/l (Figure S2). As shown in Figure 3C, the mRNA levels of *fumAC* significantly increased in FA feedstocks conditions. Thus, *fumAC* might be the primary enzyme to supply L‐malate and maintain the efficiency of the TCA cycle, which might be essential in FA conditions. Next, we carried out a two‐stage bioconversion using BA93 for β‐alanine production at 1 l fed‐batch level with soybean oil as feedstocks. Unfortunately, the cell growth of BA93 was significantly slower in the fed‐batch cultivation (Table S3). The FA utilization during the bioconversion stage was also seriously impeded (Figure S5). The β‐alanine yield could not be accurately determined because of insoluble oil‐source accumulated (Figure S5). β‐Alanine production reached about 35 g/l after a total bioconversion time of 50 h (Figure 4E).

### Complete and efficient glyoxylate‐TCA cycle is necessary for fed‐batch cultivation

**Figure 5:**
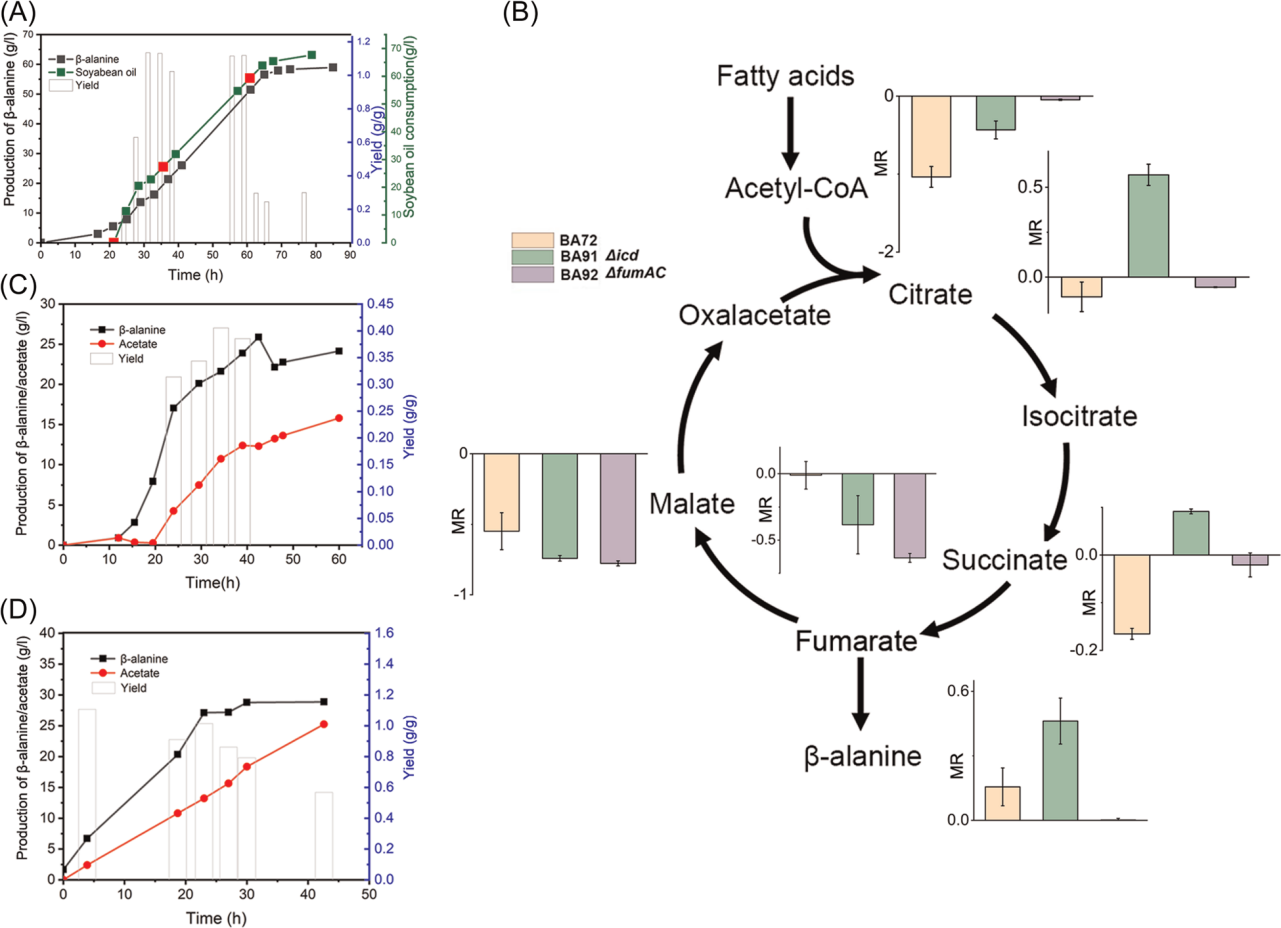
Fed‐batch β‐alanine bioconversion of different strains. (A) Bioconversion of BA72. Filled black square: β‐alanine production; filled green square: soybean oil consumption; filled red square: 2 g/l yeast extract was added; open column: yield. (B) Comparison of metabolite ratio between different bioconversion stages of BA72/91/92 (25 h and 60 h). The Y axis indicated base 10 logarithm of the metabolite ratio (MR). Metabolites ratio is the relative metabolite content of 60 h/25 h. The results given are mean ± SD (*n* = 3). (C) Bioconversion of BA92. (D) Bioconversion of BA92 using off‐site strategy. Filled square: β‐alanine production; filled circle: acetate production; open column: yield.

We speculated that the total blockage of the TCA cycle might lead to undesirable results in scaled‐up cultivations. Thus, strain BA72 was used as a control to produce β‐alanine in the fed‐batch cultivation. Unlike BA93, the FAs can be efficiently consumed by strain BA72 (Figure 5A). The β‐alanine production reached 56.57 g/l (increased 51.05 g/l during bioconversion stage) after total bioconversion time of 65 h.

To further investigate the change of metabolic flux in the fed‐batch cultivation, the intracellular levels of TCA cycle intermediates were determined using UPLC‐MS/MS (Figure 5B). When *icd* was knocked out (BA91), a large amount of citrate was accumulated after bioconversion for 60 h. This result implied that the oxidative branch of TCA might be still necessary to maintain the glyoxylate‐TCA cycle. Surprisingly, the strain BA92, with only *fumAC* knocked‐out, could even produce β‐alanine with 1.10 ± 0.05 g/g yield in the flask cultivation (Figure S4), but only produced β‐alanine with a yield of 0.4 g/g in the fed‐batch mode. The titer of β‐alanine was only about 25.88 g/l in 43 h. Meanwhile, a large amount of acetate was accumulated to 13.62 g/l (Figure 5C). In an optimized bioprocess (off‐site bioconversion strategy), in which the second stage was carried out using collected cells in a fresh medium, the β‐alanine yield of BA92 could be restored to 1.1 g/g in bioconversion for 20 h (total fermentation time of 40 h). But acetate was still accumulated and the yield was gradually decreased after 23 h (Figure 5D). We speculated that acetyl‐CoA could not be efficiently directed into the TCA cycle in BA92. As shown in Figure 5B, accompanied with the accumulation of acetate, the levels of malate and fumarate in BA92 were significantly decreased after bioconversion for 60 h. These results clearly indicated that the accumulation of acetate was due to the inefficient supply of OAA caused by blocking the reduced‐branched TCA cycle, which impeded the entry of acetyl‐CoA into the TCA cycle. Taken together, an efficient glyoxylate‐TCA cycle is necessary to maintain the metabolic flux from FAs toward β‐alanine product in the fed‐batch cultivation. An alternative strategy rather than blocking the TCA cycle should be implemented to further improve β‐alanine yield.

### β‐alanine production is significantly improved by reprogramming the glyoxylate‐TCA cycle

**Figure 6:**
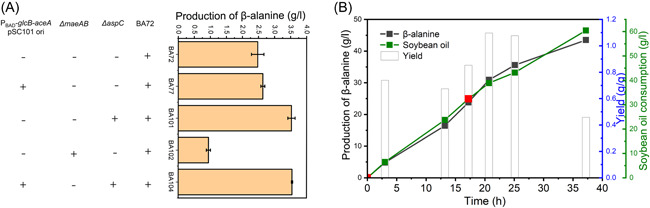
Modification of TCA targets. (A) Production of β‐alanine for different strains. BA101: *aspC* knockout; BA102: *maeAB* knockout; BA104: BA101 containing psB1a*‐glcB*‐RBS‐*aceA*. β‐alanine production was determined after bioconversion for 3 h using palmitic acid as feedstocks. (B) Bioconversion of BA104 strain. Filled black square: β‐alanine production; filled green square: soybean oil consumption; open column: yield; filled red square: 2 g/l yeast extract was added.

According to above results, an important issue is to improve OAA supply and promote the reprogramming of the glyoxylate‐TCA cycle. A series of potential metabolic targets was then investigated. First, among the targets within or related to the reductive branch TCA cycle, knocked out of *aspC* is the most effective. In the *aspC* knocked‐out strain BA101, β‐alanine production could be significantly increased to about 3.53 ± 0.11 g/l in the flask cultivation (Figure 6A). Furthermore, citrate accumulation in BA91 (Figure 5B) implied that the metabolic flux through the glyoxylate shunt should be further improved. To this end, the genes related to the glyoxylate shunt were re‐targeted. The strain BA104, in which the expression of *glcB* and *aceA* were enhanced based BA101, could produce more β‐alanine. In a fed‐batch cultivation, the titer of BA104 could reach 35.57 g/l after 25 h of bioconversion (Figure 6B), with a productivity of 1.42 g/l/h, higher than that of BA72 (1.03 g/l/h). And no acetate accumulation was found. GlcB seems to play a primary role in the glyoxylate shunt under this condition, in line with the transcription result (Figure 3C). It was also noteworthy that knocked out *maeAB* significantly decreased β‐alanine production (BA102) (Figure 6A). This result confirmed that a dynamic balance of malate‐oxaloacetate‐pyruvate plays a key role in the regulation of TCA cycle flux during β‐alanine production.

### Relieving oxygen damage in the fed‐batch cultivation can improve cell viability and β‐alanine production

**Figure 7:**
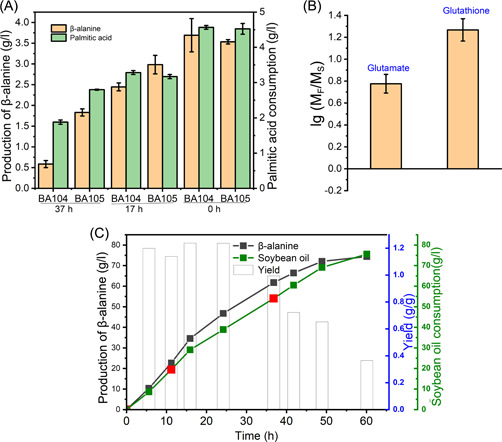
Relieving oxygen damage in the fed‐batch cultivation. (A) Relieving β‐alanine production ability in BA105 strain compared with BA104. Cells were harvested in both early (17 h) (0 h as control) stage and late stage (37 h) from fed‐batch culture and the β‐alanine production was investigated in the flask by bioconversion for 3 h with palmitic acid as feedstocks. 0 h was the time of the start of the bioconversion phase in fed‐batch cultivation. 17 h and 37 h were the time of bioconversion for 17 h and 37 h separately. (B) FAs utilization resulted in oxidative stress to BA104. The Y‐axis is the logarithm of glutathione and glutamate levels in fast‐FA utilization (M_F_) stage divided by that in slow‐FA utilization (M_S_) stage. The results given are mean ± SD (*n* = 3). (C) Bioconversion of BA105 strain. Filled black square: β‐alanine production; filled green square: soybean oil consumption; open column: yield; filled red square: 2 g/l yeast extract was added.

During the late stage of bioconversion (37h), the β‐alanine yield of BA104 was decreased significantly (Figure 6B). We further tested the cell viability in fed‐batch culture. Cells were harvested in both early (17 h) and late stage (37 h) and the β‐alanine production was investigated in the flask. It was shown that cells from a late stage produce very low amount of β‐alanine (Figure 7A). According to the results of intracellular metabolite analysis, the level of glutathione, a key metabolite in oxidative stress, increased by about 21.84 folds in the fast‐FA utilization stage (Figure 7B), and the level of glutamate, which might related to the synthesis of glutathione, also increased by about 6 folds. Glutathione plays an important role in maintaining intracellular redox balance and preventing damage caused by reactive oxygen species. This result implied that the decrease of cell viability and FA utilization (Figure S6) might be related to oxygen damage, most likely caused by the FA metabolism. Thioredoxin/glutathione peroxidase (BtuE) and glutathione reductase (Gor) are the main enzymes involved in the glutathione cycle. Thus, the expression of *btuE* and *gor* genes was enhanced by replacing native promoters with the medium‐strong promoter P119. The new strain BA105 was thus obtained and fed‐batch bioconversion of BA105 was then carried out. The results indicated that the final β‐alanine production reached approximately 72.05 g/l after bioconversion for 49 h (total bioconversion time of 70 h), with a maximum yield of 1.24 g/g (Figure 7C) and overall yield of 1.04 g/g (49 h). And the strain BA105 still retained the β‐alanine production ability during the late stage of bioconversion (Figure 7A). Taken together, the study here demonstrated a novel β‐alanine bioproduction route using FA feedstocks, obtaining the highest titer and yield reported so far.

## DISCUSSION

In this study, a carbon‐efficient glyoxylate‐TCA cycle was developed using FAs as feedstocks. By systematical modification of TCA cycle and glyoxylate shunt targets and balancing metabolic flux, β‐alanine, an important TCA cycle‐derived chemical, could be produced with high yield. Previously, two β‐alanine production routes were developed using glucose as feedstock (Table 1). Song et al. reported the production of β‐alanine from glucose through the glyoxylate shunt combined with the PEPC pathway to supply L‐aspartate resulted in 32.3 g/l and an overall yield of 0.135 g/g glucose^[^ ^6^ ^]^. Piao et al. developed a biosynthesis pathway that directly uses the PEPC pathway and blocks glycolysis to maximize the supply of OAA and L‐aspartate. Through this pathway, 37.7 g/l of β‐alanine could be produced with an overall yield of 0.425 g/g glucose^[^ ^5^ ^]^. Although the pathway was further optimized, the titer and productivity remained low^[^ ^22^ ^]^. In this study, β‐alanine could be efficiently produced with a titer of 72.05 g/l. A yield of 1.10 g/g (Figure S4) was obtained in the flask cultivation using palmitic acid as the feedstock, and the overall yield of 1.04 g/g was achieved in the fed‐batch cultivation using soybean oil as the feedstock (composition similar to tripalmitin). These production metrics are the highest reported so far.

**Table 1:** β‐Alanine *de novo* synthesis by different biological methods.

Pathway	Strain	Strategy	Production (g/l)	Yield (g/g)	Feedstock	Reference
PEPC	*E. coli*	Whole‐cell biotransformation	37.7	0.425	Glucose	^[^ ^5^ ^]^
Glyoxylate shunt + PEPC	*E. coli*	Fed‐batch fermentation	32.3	0.135	Glucose	^[^ ^6^ ^]^
PEPC	*E. coli*	Fed‐batch fermentation	1.07		Glycerol	^[^ ^21^ ^]^
PEPC + TCA	*E. coli*	Fed‐batch fermentation	43.12	ND	Glucose	^[^ ^22^ ^]^
Glyoxylate shunt	*E. coli*	Whole‐cell biotransformation	72.05	1.04	Soybean oil	This study

ND, not determined.

Our results indicated that improving the metabolic flux toward the glyoxylate shunt is of primary importance for β‐alanine biosynthesis. As shown in our study, glyoxylate shunt can be autonomously‐activated in response to FAs, which provides a favorable metabolic flux toward β‐alanine biosynthesis. Improving glyoxylate shunt flux by blocking oxidative‐branch of TCA cycle was also evaluated. Knocking out *sucA* or *icd* can confer higher yield in flask cultivations, but lead to severe growth inhibition and impede FA utilization (Figure S3). Although the strategy of blocking the TCA cycle has been successfully applied to improve the production of TCA cycle intermediates‐derived chemicals, such as succinate and 5‐aminolevulinic acid^[^ ^29^, ^30^ ^]^, this strategy seems not suitable in FA feedstocks. The oxidative‐branch of TCA cycle might be essential to adapt to FA utilization conditions. The use of FA feedstocks results in high yield of β‐alanine without block of the TCA cycle, indicating FAs can be an ideal carbon source to produce other TCA cycle‐derived chemicals. Furthermore, FA feedstocks enable higher ATP and NAD(P)H supply than glucose (Figure S7). This will be favorable for the biosynthesis of series of chemicals.

Because in the glyoxylate‐TCA cycle, the C4 TCA intermediates served as precursors for both β‐alanine production and TCA cycle reprogramming, the metabolic balance between these two processes is thus a key issue. Previously, it was shown *fum* is a key target to improve metabolic flux toward β‐alanine production when using glucose as feedstock^[^ ^6^ ^]^. However, the situation is different under FA feedstocks conditions. Although β‐alanine production could be enhanced by the deletion of *fumAC* in the flask cultivation, but totally deleting *fumABC* significantly impeded β‐alanine production. Furthermore, during the fed‐batch process, a large amount of acetate was accumulated in *fumAC* knocked out strain BA92. We speculated totally blocking *fum* might decrease the efficiency of glyoxylate‐TCA cycle. As implied in our metabolite analysis during the fed‐batch process, knocking out *fumAC* leads to decreasing the reductive‐branch TCA cycle metabolites, thus might inhibit the reprogram of the TCA cycle and lead to acetate accumulation. Coinciding with these results, recovering the *fum* genes and further knocking out *aspC* to increase the supply of reductive‐branch TCA intermediates could improve the efficiency of the glyoxylate‐TCA cycle and β‐alanine production.

Other metabolic regulations and responses may also affect FA metabolism and β‐alanine production. As FA metabolism could generate large amounts of NADH during β‐oxidation, respiration chain and oxygen might be necessary for NADH recycling during fast FA utilization. In line with this, obvious oxidative stress and significant cell damage were observed in the fed‐batch process. Furthermore, acetyl‐CoA is almost the only precursor for all cellular metabolism, including metabolite supply and energy generation. When using FAs as sole carbon, it is also of great importance to ensure the supplemental biosynthesis of other necessary key metabolic precursors. This proposal can be supported by the fact that knocking out *maeAB*, which is related to the balance of the pyruvate‐OAA‐malate cycle, leads to the decrease of β‐alanine production. In the future, an optimized bioconversion process at a large‐scale level is needed to balance cell metabolism and β‐alanine production in FAs. The utilization of different kinds of FA/oil feedstocks is another issue to be considered. Indeed, the β‐alanine production is lower when using myristic acid (C14) and lauric acid (C12) substrates (Figure S8). Enzymes responsible for short‐chain FAs utilization need to be improved in further study.

Taken together, our study extensively characterized the key factors that influenced the production of TCA cycle‐derived chemicals from FA feedstocks. The higher metrics of β‐alanine production from FAs provide a competitive route of β‐alanine production, as well as other important chemicals. Furthermore, utilization of all kinds of cheap oils as alternative feedstocks will be beneficial to resolve the environmental and carbon recycling issues.

## MATERIALS AND METHODS

### Chemicals and reagents

DNA polymerase, Gibson kits, and T4 DNA ligase purchased from Tsingke Biological Technology or Fisher Scientific were used for the plasmid construction. β‐alanine standards and other organic acids were purchased from Sigma‐Aldrich. Other chemicals and reagents were purchased from Shanghai Sheng Gong Biochemical Co. Ltd. for high‐performance liquid chromatography (HPLC) analysis.

### DNA manipulations

All chromosomal manipulations were carried out using λ‐red‐mediated homologous recombination^[^ ^31^ ^]^. All plasmids were constructed using the Gibson DNA assembly method^[^ ^32^ ^]^ and sequenced by Tsingke Biological Technology. If necessary, heterologous genes were synthesized by Genewiz with codon optimization for *E. coli*. The resistance markers were eliminated using the plasmid pSB1s‐Cre or pCP20 according to the Cre‐lox or FRT‐FLP system. For chromosomal promoter replacement, the CPA1 promoter, 119 promoters were amplified from pSL91k and pSLCPA1k, respectively. The DNA fragments used for λ‐red recombination were overlapped with about 500 bp of the upstream/downstream region of the target genes. The adaptive laboratory evolution process (GREACE) was performed according to the methods in the literature^[^ ^33^ ^]^. Primers used in this study are listed in Table S1.

### Bacterial strains and culture conditions

*E. coli* DH5α was used for plasmids construction. For these assays, *E. coli* strains were grown at 37°C, 220 rpm in Luria‐Bertani (10 g/l tryptone, 5 g/l yeast extract, and 10 g/l NaCl) medium. When necessary, the following antibiotics were used : 100 μg/ml ampicillin, 50 μg/ml streptomycin, or 50 μg/ml kanamycin. Overnight cultures were then inoculated with 1% inoculum into an auto‐induction medium and cultured at 30 °C, 220 rpm, for 16 h for protein expression. The medium included 1% tryptone, 0.5% yeast extract, 25 mM KH_2_PO_4_, 25 mM Na_2_HPO_4_, 50 mM NH_4_Cl, 5 mM Na_2_SO_4_, 2 mM MgSO_4_, 0.2 × trace metals, 0.5% glycerol, 0.05% glucose, 0.2% L‐arabinose and antibiotics when necessary^[^ ^34^ ^]^. The whole‐cell bioconversion for β‐alanine produced from FAs was conducted using M9 medium with FAs as feedstocks. M9 medium contained 10 g/l NH_4_Cl, 12.8 g/l Na_2_HPO_4_·7H_2_O, 3 g/l KH_2_PO_4_, 0.5 g/l NaCl, and 5 g/l palmitic acids (4 g/l Brij 58 was added to be emulsified). Cell mixtures were suspended in 3 ml (OD_600_ = 30) in 50 ml test tube for aerobic conditions, and 500 μl (OD_600_ = 30) in 1.5 ml centrifuge tube for anaerobic conditions. The bioconversion reactions were performed at 37°C, 200 rpm for 3 or 24 h. The strains and plasmids used in this study are listed in Table S2. Each bioconversion was repeated at least three times.

### Fed‐batch bioconversion

Monoclonal colony was picked up from LB agar plates with antibiotics and inoculated to 5 ml liquid LB medium with antibiotics to prepare the seed culture. All the seed culture was then transfered to 100 ml liquid LB medium until OD_600_ reached 2.0. 10 ml culture was then transfered to 1 l fermenter containing 500 ml CD medium (10 g/l glucose,14 g/l KH_2_PO_4_, 4 g/l (NH_4_)_2_HPO_4_, and 1.8 g/l citric acid monohydrate). 2 g/l L‐arabinose was added to the fermenter when the initial glucose was nearly exhausted. In the biomass accumulation stage, glucose was used as the main feedstock until the OD_600_ reached 60. Then glucose was replaced by FAs (soybean oil). The pH was automatically controlled at 7.0 with the addition of ammonia, and the agitation speed and airflow rate were correlated during fermentation to control the dissolved oxygen (DO) concentration.

Two bioconversion strategies were used. For the two‐stage bioconversion strategy, when OD_600_ reached 60 during the biomass accumulation stage, the cultures were directly fed with soybean oil (plus 15 mg/l lipases) supplemented with 2 g/l yeast extract when DO reached 50%, and the total amount of yeast extract did not exceed 5 g/l. The composition of soybean oil is glycerol moiety (about 11%) and different FA moieties (mainly oleic acid moiety (45.76%), linoleic acid moiety (36.91%), palmitic acid moiety (13.51%), and stearic acid moiety (3.74%)). For the off‐site bioconversion strategy, induced cells were collected by centrifugation at 8000 rpm, 4°C, 30 min, and then suspended in 500 ml bioconversion M9 medium. The medium contained 12.8 g/l Na_2_HPO_4_·7H_2_O, 3 g/l KH_2_PO_4_, 0.5 g/l NaCl, and 1 g/l NH_4_Cl. The pH was controlled at 7.0 via addition of ammonia. One representative data from three reproducible experiments is shown.

Strain BA91 and BA93 (*icd* knockout) were performed using a modified medium containing 29.03 g/l glutamate for cell growth and enzyme induction.

### Analytical methods

The optical density was measured by Ultrospec 3000 spectrometer at 600 nm. HPLC was used to identify β‐alanine concentrations, and the analysis conditions are as follows: the analysis was controlled at 40°C; mobile phase A was 0.1% formic acid, mobile B was acetonitrile and the ratio was 65:35, and the flow rate was controlled at 0.5 ml/min. To identify β‐alanine, dinitrofluorobenzene (DNFB) and 0.1 M NaHCO_3_ were added to samples by derivatization at 60°C, 1 h after that 0.1% formic acid in water was used to terminate reaction and 0.22 μM PES membrane (Jinteng) was used to be filtered. The column was Ascentis Express ‐C18 2.7 μm (4.6 × 250 mm). The DAD detector at 360 nm was used. Other compounds (glucose and organic acids) were identified by HPLC (Agilent 1260 series), and the Aminex HPX‐87H column (300 × 7.8 mm, Bio‐Rad, USA) was used. The mobile phase was 5 mM H_2_SO_4_ in water at 55°C and the flow rate was 0.6 ml/min. All data were obtained from three replicates. A gas chromatograph (GC, 7890A) was used (Agilent Technologies, USA) to identify FA concentrations with a flame ionization detector (FID). The reagent samples were converted to FAME (FA methyl esters) to be analyzed by GC, and each 300 μl FAs sample without supernatant, mixing with 500 μl 10% (V/V) H_2_SO_4_ in methanol reacted at 60°C for 20 min, then the mixture was mixed by 500 μl hexane, then vortexed vigorously, the organic and aqueous layers were separated by centrifuging at maximum speed, and the organic layer was measured by GC. The program was as follows: 150°C, 5 min, ramping up to 170°C slowly and the heating rate of 3°C/min, held at 170°C for 5 min, increased up to 210°C further and the increase rate of 3°C/min, held at 210°C for 5 min, with an HP‐88 column (60 m × 0.25 mm, 0.2 μm film thickness). The yield was calculated as the ratio of the amount of β‐alanine produced to the palmitic acid consumed. The overall yield was determined from the total amounts of β‐alanine production and soybean oil consumption during bioconversion. The productivity was calculated as β‐alanine production per hour during the initial fast‐bioconversion phase.

Strain FA09 was cultured in M9 salt with the carbon resources FAs or glucose medium separately in 500 ml flasks, cultured to mid‐exponential growth phase and harvested by centrifugation, then 0.85% NaCl solution was added to the medium to wash for three times, and liquid nitrogen was used to be frozen immediately and stored at −80°C. Samples were sent to Vazyme Biotech Co., Ltd. for RNA extraction and transcriptome sequencing analysis (Nanjing, China).

UPLC‐MS/MS was used to determine the intracellular metabolite concentrations. Cells from late stage and early stage in fed‐batch cultivation were used for metabolite analysis. Cells were chilled on ice, and collected at 13,000 rpm, 4°C for 10 min by centrifugation, ice‐cold 0.85% NaCl solution was added to wash for twice, and then 400 μl (OD_600_ = 90) of 80% (vol/vol) methanol was added to the mixture for preventing any further metabolism and sonicated^[^ ^35^ ^]^. The UPLC‐MS/MS was carried out by MFTC‐PS (Metabolomics Facility at the Technology Center for Protein Sciences) of Tsinghua University.

## CONFLICTS OF INTERESTS

The authors declare no conflicts of interests.

## ETHICS STATEMENT

Not Applicable.

## AUTHOR CONTRIBUTIONS

Yingchun Miao, Jiao Liu, Xuanlin Wang, and Bo Liu completed most of the experiments. Yong Tao conceived the research plan, supervised the experiments, and participated in experimental design. Yong Tao, Weifeng Liu, Bo Liu, and Yingchun Miao wrote this article. All authors reviewed and approved this submission.

## Supporting information

Supporting information.

## Data Availability

Data supporting this study are available within the paper and its Supplementary Information files. Source data are provided with this paper.
