# Supplementary material for: Fatty acid feedstocks enable a highly efficient glyoxylate‐TCA cycle for high‐yield production of β‐alanine
Source: mLife. 2022 Jun 19;1(2):171–82. doi: 10.1002/mlf2.12006 (PMC10989975; doi:10.1002/mlf2.12006)
Supplement: Supplementary file 1 — Supporting information. [file MLF2-1-171-s001.docx]

**Supplementary information**

Table S1 Primers used in this study

| **name** | **sequence** | **application** |
| --- | --- | --- |
| *TcpanD*-NcoI | GCTAACAGGAGGAATTAACCATGCCAGCAACCGGTGAGGATCAGGA | pXB1K/ pSB1K/ pLB1K / pAB1K -*TcpanD* |
| *TcpanD*-XhoI | GTACCAGATCTACCCTCGAGTTAAAGGTCGCTGCCCAGACGTTC |  |
| *AspA*- NcoI | GCTAACAGGAGGAATTAACCATGTCAAACAACATTCGTATCGAA | pSB1a-*aspA* |
| *AspA*- XhoI | GTACCAGATCTACCCTCGAGTTACTGTTCGCTTTCATCAGTATAG |  |
| *AspC*- NcoI | GCTAACAGGAGGAATTAACCATGTTTGAGAACATTACCGCCGCTC | pSB1a-*aspC* |
| *AspC*- XhoI | GTACCAGATCTACCCTCGAGTTACAGCACTGCCACAATCGCTT |  |
| *RocG*-*aspC*-FP | AGCGGCGGTAATGTTCTCAAACATAGGAGATGTCAGCAAAGCAAGTC | pSB1a-*aspC*-*RocG* |
| *RocG*- XhoI | GTACCAGATCTACCCTCGAGTTAGACCCATCCGCGGAAAC |  |
| *icd*-Lox71-F | AGGACGCAAACGCATATGCAACGTGGTGGCAGACGAGCAAACCAGTAGCGCTCGAAGGAGAGGTGATCTCGAGAATATCCTCCTT | *icd*-Knockout |
| *icd*-Lox66-R | GCAGCCTAACAAAAAACAACGGGAGCGTTACGCTCCCGTTAATAAATTTAACAAACTACGGCATAGCTTCATAACTTGGTATAGC |  |
| *SucA*-Lox71-F | ACAAGTCATTACCCGTAGGCCTGATAAGCGCAGCGCATCAGGCGTAACAAAGAAATGCAGGAAATCTCTCGAGAATATCCTCCTT | *SucA*-Knockout |
| *SucA*-Lox66-R | CATCGGCTACGGATTCAGGCAGGTCAGGGACCAGAATATCTACGCTACTCATTGTGTATCCTTTATTAGCTTCATAACTTGGTATAGC |  |
| *FumA*-250F | TTCGCCAAAGCGTTTGGCAACGGCC | *FumAC*-Knockout |
| *FumC*-250R | GCAAAGAAAACTGGAGCGAGAGTAT |  |
| *IclR*-250F | CCATACCGCCGTCCAGCACCAGA | *IclR*-Knockout |
| *IclR*-250R | TATGATTACAACTAAAGAAATATCAT |  |
| *FadR*-250F | ATGAGGAAAATTAACGGGTTTACG | *FadR*-Knockout |
| *FadR*-250R | GGAACGTGGATTTGCGCGGCGAC |  |
| *FadD*-Lox71-F | TTCTGCATTCTTACGGTAAAGATAAAAATAAATAGTGACGCGCTTCGCAACCTTTTCGTTGGGTAATCTCGAGAATATCCTCCTT | *FadD*-promoter PCPA1 |
| *FadD*-CPA1-R | ATAACGGTCAGGGTTGATCTCCGTCGGAACGTCCGCGGGATAACGGTTAAGCCAAACCTTCTTCAATATATCTCCTTCTTAAAAG |  |
| *FumB*-250-F | CTACGGTTATTACATCCTGCCGAC | *FumB*-Knockout |
| *FumB*-250-R | CAGTAGCTGCACGACAATAATCAC |  |
| *AceB*-Lox71-F | AATAAATTTTATTCATATTGTTATCAACAAGTTATCAAGTATTTTTAATTAAAATGGAAATTGTTTTCTCGAGAATATCCTCCTT | *AceBAK*-promoter P119 |
| *AceB*-119-R | TTGCTTCTCCTGCTCGCCATACGGCCTTGTGAAAGCCAGTTCATCGGTTGTTGTTGCCTGTTCAGTCATGGCTAGTATTTCTCCTC |  |
| *BtuE*-Lox71-F | GTCACCGCAACGTTAGGTGCGCCGGTGTTTATCTGGTTATTGTTAAAAGCAGGACGTTAGCCGCAATCTCGAGAATATCCTCCTT | *BtuE*-promoter P119 |
| *BtuE*-119-R | GAACTTCTCCAGCGTGGTCACTTCACCGTCGATATCTTTCACTACGGTCGTCAGAATGGAATCTTGCATGGCTAGTATTTCTCCTC |  |
| *gor*-Lox71-F | AGCCATTGCTGGCACCTATTACGTCTCGCGCTACAATCGCGGTAATCAACGATAAGGACACTTTGTTCTCGAGAATATCCTCCTT | *gor*-promoter P119 |
| *gor*-119-R | CGCGCGGTTGATGGAGGCGATACCGCCGCTGCCGCCGCCGATGGCGATGTAATCATAGTGTTTAGTCATGGCTAGTATTTCTCCTC |  |
| *AspC*-200F | GAAAGACGTAGAAGGTATCGGTG | *aspC*-knockout |
| *AspC*-200R | TGATATTTATTCCGGGTCACGGAC |  |
| *aceB*-Nco1-F | GCTAACAGGAGGAATTAACCATGACTGAACAGGCAACAACAACC | *aceBA* overexpression |
| *aceA*-xho1-R | GTACCAGATCTACCCTCGAGTTAGAACTGCGATTCTTCAGTGGA |  |
| *glcB*-Nco1-F | GCTAACAGGAGGAATTAACCATGAGTCAAACCATAACCCAGAGC | *GlcB-aceA* overexpression |
| *GlcB*-RBS-R | AATTTGTTGTGTACGGGTTTTCATCCTCCTTTAATGACTTTCTTTTTCGCGTAAACG | *aspC*-knockout |
| RBS-*aceA*-F | CGTTTACGCGAAAAAGAAAGTCATTAAAGGAGGATGAAAACCCGTACACAACAAATT |  |
| *MaeA*-200F | AAATCGAACCGTCAGGCACGTCATA | *MaeA*-Knockout |
| *MaeA*-200R | TAATGAGGTGACACCTGGACCCAC |  |
| *BspanD-* NcoI | GCTAACAGGAGGAATTAACCATGGGTCACCACCACCACCACCACA | pSL91k-*BspanD* |
| *BspanD-* XhoI | GTACCAGATCTACCCTCGAGTTACAGGATGGTGCGAGCCGGTT |  |
| *MaeB*-200F | TTCATAGGAAATACTCCTTGAA | *MaeB*-Knockout |
| *MaeB*-200R | GGCACAAATTCCTGAATGATGCG |  |
| *ptsG*-200F | ATCCGTTGAATGAGTTTTTTTAAAG | *ptsG*-Knockout |
| *ptsG*-200R | CCGAGCAAGGTAGCTACGCGCT |  |
| *galR*-200F | ACCCGAAGTTGACTTCACCTATCAA | *galR*-Knockout |
| *galR*-200R | CACGCGCCAACGGTGGAAACCGT |  |
| *glk-* NcoI | GCTAACAGGAGGAATTAACCATGACAAAGTATGCATTAGTCGGT | pSL91k-*glk* |
| *glk-* XhoI | GTACCAGATCTACCCTCGAGTTACAGAATGTGACCTAAGGTC |  |

Table S2. Strains and plasmids used in this study.

| **strains** | **Relevant genotype** | **Reference** |
| --- | --- | --- |
| *E. coli* BW25113/F׳ | *rrnBT14 ΔlacZWJ16 hsdR514 ΔaraBADAH33 ΔrhaBADLD78* [*F׳ proAB lacIqZΔM15 Tn10 (Tetr)*] | CGSC |
| FA09 | 1. *coli* BW25113/F׳, Δ*fadR*, PCPA1-fadD, P119-fadL, Δ*sthA*, PCPA1-*pntAB* | [17] |
| BA20 | FA09 containing pSB1K-*TcpanD* | This study |
| BA21 | FA09 containing pLB1K-*TcpanD* | This study |
| BA22 | FA09 containing pXB1K-*TcpanD* | This study |
| BA23 | FA09 containing pAB1K-*TcpanD* | This study |
| BA30 | FA09 P119- *BspanD* | This study |
| BA31 | BA30 Δ*iclR* | This study |
| BA32 | BA30 PCPA1-*aspA* | This study |
| BA33 | BA32 Δ*iclR* | This study |
| FG00 | *E. coli* BW25113/F׳, Δ*iclR*, Δ*ptsG,* Δ*galR,* P119*-glk,* P119- *BspanD,* PCPA1-*aspA* | This study |
| BA34 | BA32 P119-*glcB*-RBS-*aceA* | This study |
| BA35 | BA32 P119-*aceB*-RBS-*aceA* | This study |
| BA36 | BA35 Δ*iclR* | This study |
| BA37 | BA31 Δ*sucA* | This study |
| BA38 | Evolved from BA37 | This study |
| BA39 | BA33 Δ*icd* | This study |
| BA40 | BA33 Δ*fumAC* | This study |
| BA41 | BA39 Δ*fumAC* | This study |
| BA42 | BA41 Δ*fumB* | This study |
| BA43 | BA43 Δ*aspC* | This study |
| BA44 | BA43 Δ*maeAB* | This study |
| BA45 | BA43 P119-*btuE* P119-*gor* | This study |
|  |  |  |
| BA52 | BA30 containing pXB1K-*TcpanD* | This study |
| BA53 | BA30 containing pSB1a-*aspA*, pXB1K-*TcpanD* | This study |
| BA62 | BA32 containing pXB1K-*TcpanD* | This study |
| BA72 | BA33 containing pXB1K-*TcpanD* | This study |
| BG22 | FG00 containing pXB1K-*TcpanD* | This study |
| BA73 | BA34 containing pXB1K-*TcpanD* | This study |
| BA74 | BA35 containing pXB1K-*TcpanD* | This study |
| BA75 | BA36 containing pXB1K-*TcpanD* | This study |
| BA76 | BA75 containing pSB1a- *aceB*-RBS-*aceA* | This study |
| BA77 | BA75 containing pSB1a- *glcB*-RBS-*aceA* | This study |
| BA80 | BA31 containing pSB1a-*aspC*-*rocG*, pXB1K-*TcpanD* | This study |
| BA81 | BA37 containing pSB1a-*aspA*, pXB1K-*TcpanD* | This study |
| BA82 | BA37 containing pSB1a-*aspC* pXB1K-*TcpanD* | This study |
| BA83 | BA37 containing pSB1a-*aspC*-*rocG* pXB1K-*TcpanD* | This study |
| BA85 | BA38 containing pSB1a-*aspC*-*rocG* pXB1K-*TcpanD* | This study |
| BA91 | BA39 containing pXB1K-*TcpanD* | This study |
| BA92 | BA40 containing pXB1K-*TcpanD* | This study |
| BA93 | BA41 containing pXB1K-*TcpanD* | This study |
| BA94 | BA42 containing pXB1K-*TcpanD* | This study |
| BA101 | BA43 containing pXB1K-*TcpanD* | This study |
| BA102 | BA44 containing pXB1K-*TcpanD* | This study |
| BA104 | BA43 containing pSB1a-*glcB*-RBS-*aceA* pXB1K-*TcpanD* | This study |
| BA105 | BA45 containing pSB1a-*glcB*-RBS-*aceA* pXB1K-*TcpanD* | This study |
| **Plasmid** | **Description** | **Source** |
| pXB1k | araBAD promoter, p15A ori, Kan^r^ | Our laboratory |
| pSB1k | araBAD promoter, pSC101 ori, Kan^r^ | Our laboratory |
| pSB1a | araBAD promoter, pSC101 ori, amp^r^ | Our laboratory |
| pLB1k | araBAD promoter, r6k ori, Kan^r^ | Our laboratory |
| pAB1k | araBAD promoter, ColA ori, Kan^r^ | Our laboratory |
| pKD46 | Temperature sensitive vector carrying λ-Red recombinase, Amp^r^ | [35] |
| pSL91k | 119 promoter, Kan^r^ selective marker with lox71 and lox66, pSC101 ori | Our laboratory |
| pSLCPA1k | CPA1 promoter, Kan^r^ selective marker with lox71 and lox66 pSC101 ori | Our laboratory |
| pXB1k-*TcpanD* | pXB1k containing *Tribolium castaneum* *panD* | This study |
| pSB1a-*aspA* | pSB1a containing *E. coli* *aspA* | This study |
| pSB1a-*aspC* | pSB1a containing *E. coli aspC* | This study |
| pSB1a-*aspC*-*rocG* | pSB1a containing *Bacillus subtilis rocG* and *E. coli aspC* | This study |
| pSB1s-*cre* | pSB1s containing *Cre* recombinase gene, Str^r^ | Our laboratory |
| pCP20 | Temperature sensitive vector carrying FLP, Amp^r^ | [35] |

**References**:

**17.** Liu B , Xiang S , Zhao G , Wang B , Ma Y , Liu W , et al. Efficient production of 3‐hydroxypropionate from fatty acids feedstock in *Escherichia coli* . Metab Eng. 2019;51:121–30.

**35.** Datsenko KA , Wanner BL . One‐step inactivation of chromosomal genes in *Escherichia coli* K‐12 using PCR products. Proc Natl Acad Sci USA. 2000;97:6640–5.

Table S3 cell growth of *icd* knockout strains in the fed-batch cultivation

| OD600  Strains | 0h | 13h | 21.4h |
| --- | --- | --- | --- |
| BA72 | 0.01 | 31 | 68 |
| BA93 (totally 29 g/L L-glutamate) *icd* knockout | 0.01 | 25.4 | 60.8 |


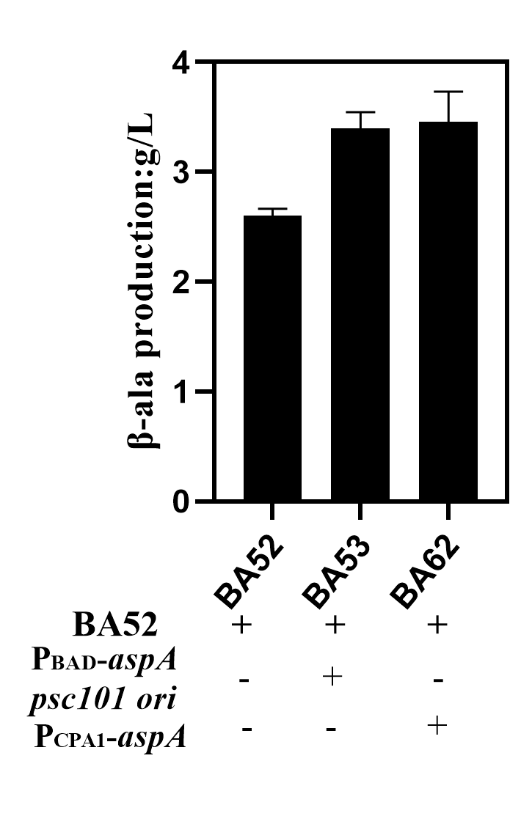


Supplementary Fig.S1. β-alanine production with *aspA* overexpression. BA52: without-*aspA* expressed; BA53: plasmid pSB1a-*aspA* overexpression; BA62: chromosomal *aspA* expressed.


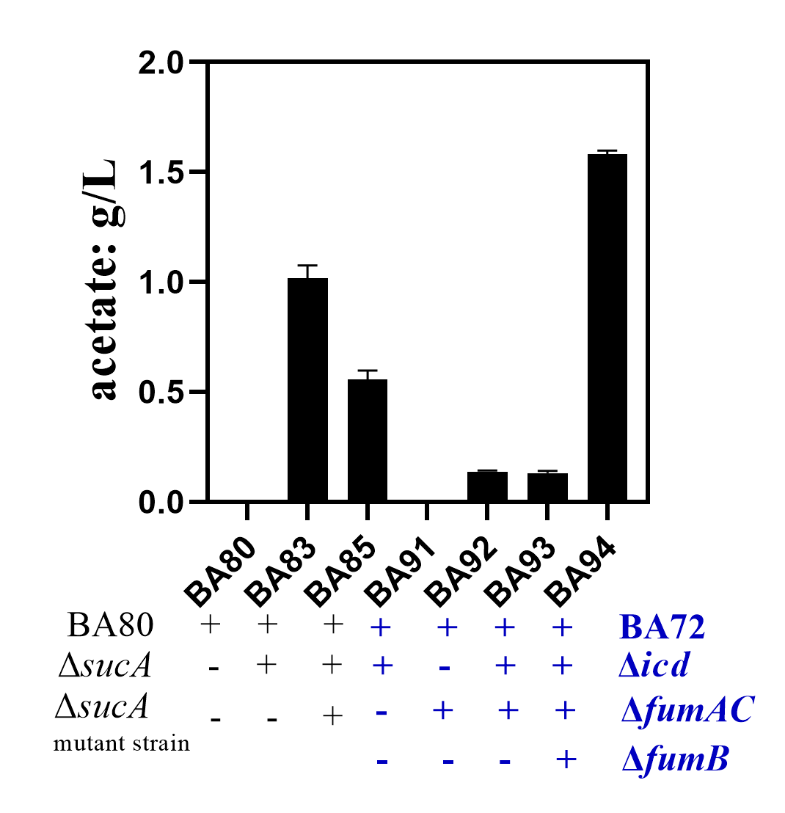


Supplementary Fig.S2. Strains with the accumulation of [by-product](javascript:;) acetate bioconversion for 24 hrs. BA83: *sucA* knockout; BA85: *sucA*-knockout evolved strains; BA91: *icd*- knockout; BA92: *fumAC*- knockout; BA93: *icd* and - *fumAC* double knockout; BA94: *icd* and – *fumACB* triple knockout.


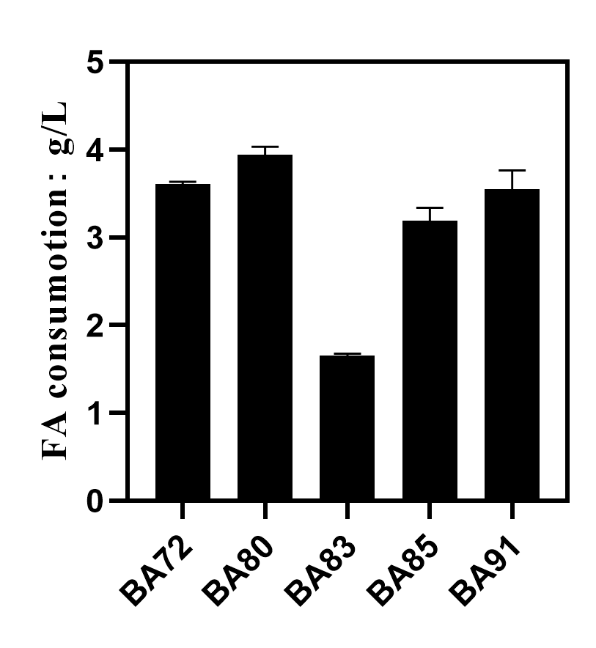


Supplementary Fig.S3. Strains with FAs utilization for 3 hrs. BA72: WT without *icd/sucA* knockout with *aspA* overexpressed; BA80: WT without *icd/sucA* knockout with *aspC-rocG* overexpressed BA83: *sucA*-knockout; BA85: *sucA*-knockout evolved strains; BA91: *icd*- knockout.


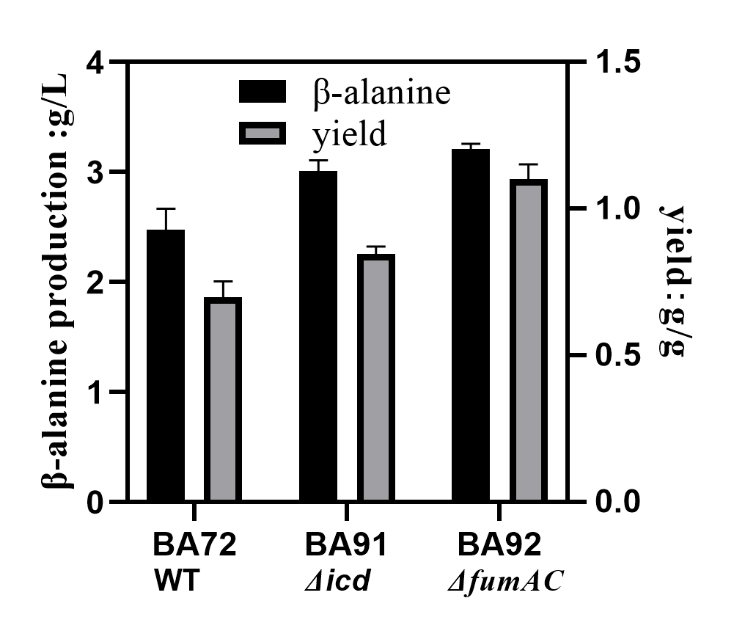


Supplementary Fig.S4. Strains with *ICD/fumAC* single knockout for 3 hrs. BA72: WT without *icd/fumAC* knockout; BA91: *icd*- knockout; BA92: *fumAC*- knockout.


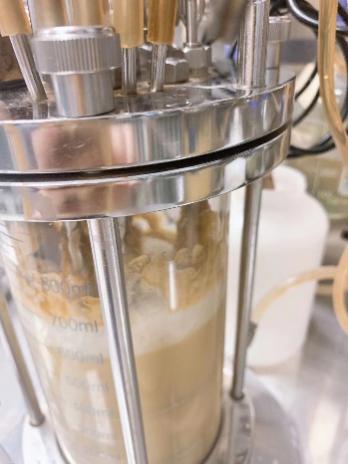


Supplementary Fig.S5. Strains with BA93 in the fed-batch cultivation for insoluble oil-source accumulation


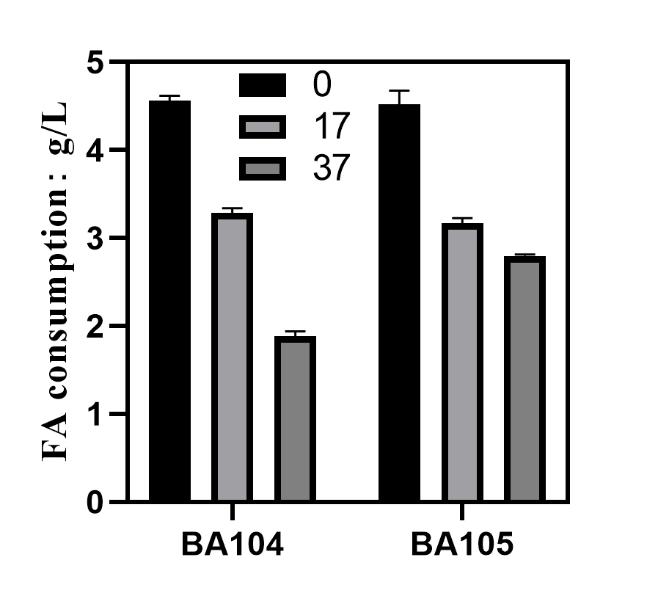


Supplementary Fig.S6. BA104 and BA105 with FAs utilization in the flask cultivation for 3 hrs from fermenters at 0 h, 17 h, 37 h, 30 OD/mL (approximately).


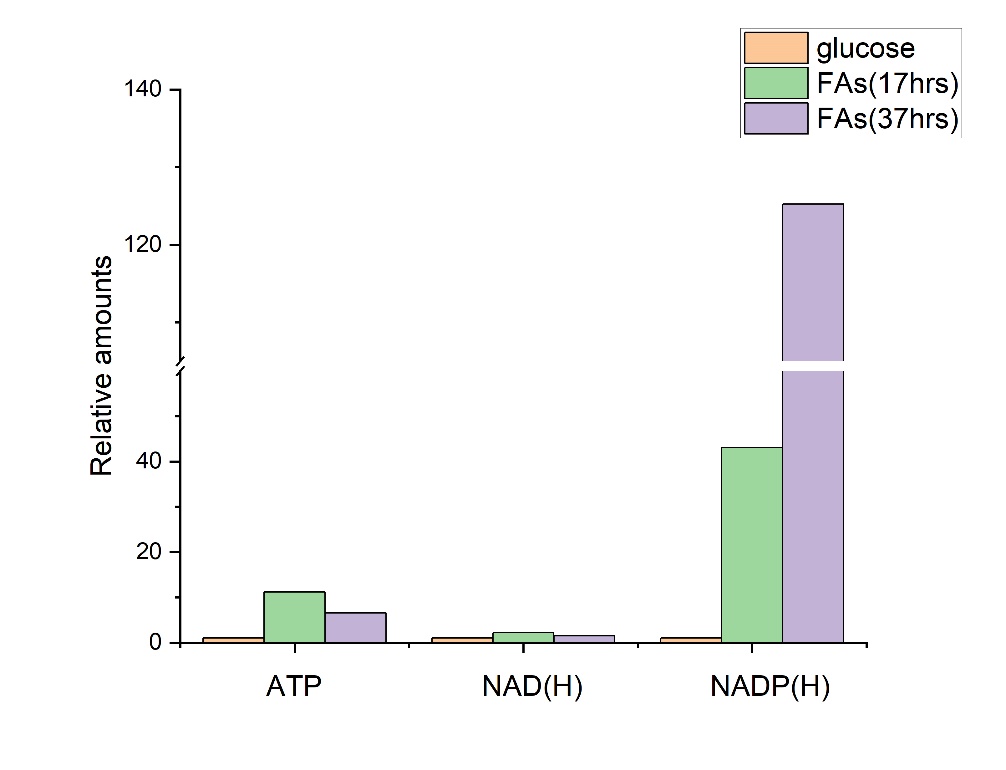


Supplementary Fig.S7. The relative amounts of ATP, NAD(H) and NADP(H) of BA105 during bioconversion from glucose or FAs (soybean oil) feedstocks at 1L level..


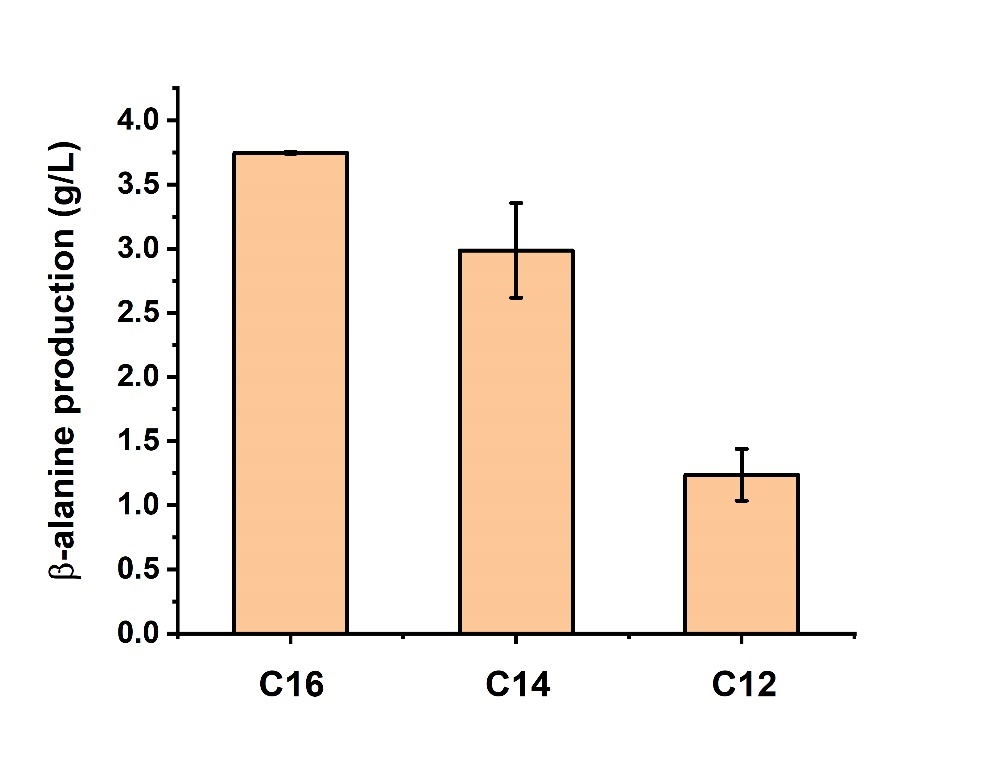


Supplementary Fig.S8. The β-alanine production using different length of FAs feedstocks (palmitic acid (C16), myristic acid (C14) and lauric acid (C12)). Whole-cell bioconversion from myristic acid (C14), palmitic acid (C16) or lauric acid (C12) was carried out in the flask cultivation for 3 hrs.
